# Supplementary material for: Comparative transcriptomics reveals divergence in pathogen response gene families amongst 20 forest tree species
Source: G3 (Bethesda). 2023 Oct 9;13(12):jkad233. doi: 10.1093/g3journal/jkad233 (PMC10700026; doi:10.1093/g3journal/jkad233)
Supplement: jkad233_Supplementary_Data [file jkad233_supplementary_data.zip › manuscript_suppl.docx]

Fig. S1. Research methodology.

Table S1. Quality of the sequence data and the number of assembled transcripts. Raw reads - total amount of reads in raw data; Effective - (Clean reads/Raw reads)*100%; Error - base error rate; Q20, Q30 - (Base count of Phred value >20 or 30) / (Total base count); GC - (G & C base count) / (Total base count)

| Sample | Raw reads | Raw data  (Gb) | Effective  (%) | Error(%) | Q20  (%) | Q30  (%) | GC  (%) | Number of transcripts |
| --- | --- | --- | --- | --- | --- | --- | --- | --- |
| *Acer rubrum* | 42516252 | 6.4 | 99.03 | 0.03 | 96.86 | 91.99 | 43.55 | 26798 |
| *Acer saccharum* | 40258404 | 6 | 99.09 | 0.03 | 96.56 | 91.48 | 44.93 | 19713 |
| *Acer spicatum* | 40951588 | 6.1 | 99.01 | 0.03 | 96.77 | 91.86 | 44.39 | 20529 |
| *Alnus incana* | 40746306 | 6.1 | 98.63 | 0.03 | 97.07 | 92.51 | 46.61 | 25900 |
| *Amelanchier laevis* | 40642302 | 6.1 | 98.61 | 0.03 | 96.81 | 91.95 | 47.28 | 28845 |
| *Betula alleghaniensis* | 40110170 | 6 | 98.77 | 0.03 | 96.75 | 91.86 | 46.77 | 21388 |
| *Betula papyrifera* | 42242750 | 6.3 | 99.14 | 0.03 | 96.87 | 92.18 | 46.57 | 24199 |
| *Cornus alternifolia* | 39167342 | 5.9 | 99.59 | 0.02 | 98.25 | 94.87 | 45 | 22372 |
| *Corylus cornuta* | 42406264 | 6.4 | 99.69 | 0.02 | 98.21 | 94.77 | 46.92 | 19459 |
| *Dirca palustris* | 39318736 | 5.9 | 99.67 | 0.02 | 98.35 | 95.11 | 48.22 | 24726 |
| *Fraxinus nigra* | 40215038 | 6 | 99.54 | 0.02 | 98.36 | 95.05 | 44.35 | 28355 |
| *Ostrya virginiana* | 40860242 | 6.1 | 99.57 | 0.02 | 98.21 | 94.8 | 47.36 | 21072 |
| *Populus grandidentata* | 42223600 | 6.3 | 99.67 | 0.02 | 98.32 | 94.89 | 44.1 | 25882 |
| *Populus tremuloides* | 40203804 | 6 | 99.23 | 0.03 | 96.95 | 92.14 | 44.81 | 25360 |
| *Prunus serotina* | 40248228 | 6 | 99.65 | 0.02 | 98.33 | 94.97 | 45.48 | 24729 |
| *Prunus virginiana* | 40791586 | 6.1 | 98.18 | 0.03 | 96.91 | 92.23 | 46.07 | 24768 |
| *Quercus rubra* | 43706072 | 6.6 | 98.05 | 0.03 | 97.41 | 93.1 | 44.47 | 25300 |
| *Ribes cynosbati* | 40106626 | 6 | 98.97 | 0.03 | 96.57 | 91.52 | 44.57 | 21203 |
| *Tilia americana* | 41811102 | 6.3 | 99.06 | 0.03 | 96.81 | 91.92 | 44.31 | 31804 |
| *Ulmus americana* | 43900232 | 6.6 | 98.69 | 0.03 | 97.7 | 93.78 | 46 | 26486 |

Fig. S2. Completeness assessment of assembled transcriptomes using BUSCO with dataset embryophyta_odb10 (a), and numbers of annotated transcripts (b).

Fig. S3. Distribution of Ks (the level of synonymous substitutions) within and between the *Populus* and *Prunus* species.

Fig. S4. GO terms enrichment in gene families with a size $\geq$5 and Ka/Ks $>$1. A_rubr *- Acer rubrum*; A_sacc - *Acer saccharum*; A_spic - *Acer spicatum*; A_inca - *Alnus incana*; A_laev - *Amelanchier laevis*; B_alle - *Betula alleghaniensis*; B_papy - *Betula papyrifera*; C_alte - *Cornus alternifolia*; C_corn - *Corylus cornuta*; D_palu - *Dirca palustris*; F_nigr - *Fraxinus nigra*; O_virg - *Ostrya virginiana*; P_gran - *Populus grandidentata*; P_trem - *Populus tremuloides*; P_sero - *Prunus serotina*; P_virg - *Prunus virginiana*; Q_rubr - *Quercus rubra*; R_cyno - *Ribes cynosbati*; T_amer - *Tilia americana*; U_amer - *Ulmus americana*. The color bars represent the *P*-values of GO enrichment test.

Fig. S5. Numbers of expanded (+) and contracted (-) gene families predicted using the program CAFE5 amongst diploid species.

Fig. S6. Numbers of expanded (+) or contracted (-) gene families attached with the KEGG term plant-pathogen interaction. Some gene families may have the same gene annotation. The dark grey cells represent the annotation terms were not found in the expanded or contracted gene families; the color bar represents the number of gene families with expansion (positive value) or contraction (negative values). C_alte - *Cornus alternifolia*; F_nigr - *Fraxinus nigra*; D_palu - *Dirca palustris*; T_amer - *Tilia americana*; A_sacc - *Acer saccharum*; A_spic - *Acer spicatum*; B_papy - *Betula papyrifera*; A_inca - *Alnus incana*; C_corn - *Corylus cornuta*; O_virg - *Ostrya virginiana*; Q_rubr - *Quercus rubra*; A_laev - *Amelanchier laevis*; R_cyno - *Ribes cynosbati.*
